# Metabolomic and Physiological Effects of a Cardiorenal Protective Diet Intervention in African American Adults with Chronic Kidney Disease

**DOI:** 10.3390/metabo14060300

**Published:** 2024-05-25

**Authors:** Meera J. Patel, Chiamaka Emerenini, Xuan Wang, Teodoro Bottiglieri, Heather Kitzman

**Affiliations:** 1 Peter J. O’Donnell Jr. School of Public Health, UT Southwestern Medical Center, Dallas, TX 75390, USA; heather.kitzman@utsouthwestern.edu; 2 College of Natural Sciences, University of Texas at Austin, Austin, TX 78712, USA; chiemerenini@utexas.edu; 3 Center of Metabolomics, Institute of Metabolic Disease, Baylor Scott & White Research Institute, Dallas, TX 75204, USA; xuan.wang@bswhealth.org (X.W.); teodoro.bottiglieri@bswhealth.org (T.B.)

**Keywords:** chronic kidney disease, hypertension, cardiovascular disease, African American adults, diet intervention, metabolomic analysis

## Abstract

Chronic kidney disease (CKD) impacts 14% of adults in the United States, and African American (AA) individuals are disproportionately affected, with more than 3 times higher risk of kidney failure as compared to White individuals. This study evaluated the effects of base-producing fruit and vegetables (FVs) on cardiorenal outcomes in AA persons with CKD and hypertension (HTN) in a low socioeconomic area. The “Cardiorenal Protective Diet” prospective randomized trial evaluated the effects of a 6-week, community-based FV intervention compared to a waitlist control (WL) in 91 AA adults (age = 58.3 ± 10.1 years, 66% female, 48% income ≤ USD 25K). Biometric and metabolomic variables were collected at baseline and 6 weeks post-intervention. The change in health outcomes for both groups was statistically insignificant (*p* > 0.05), though small reductions in albumin to creatinine ratio, body mass index, total cholesterol, and systolic blood pressure were observed in the FV group. Metabolomic profiling identified key markers (*p* < 0.05), including C3, C5, 1-Met-His, kynurenine, PC ae 38:5, and choline, indicating kidney function decline in the WL group. Overall, delivering a directed cardiorenal protective diet intervention improved cardiorenal outcomes in AA adults with CKD and HTN. Additionally, metabolomic profiling may serve as a prognostic technique for the early identification of biomarkers as indicators for worsening CKD and increased CVD risk.

## 1. Introduction

Chronic kidney disease (CKD) and hypertension (HTN) have been demonstrated as risk factors for end-stage renal disease (ESRD) and cardiovascular disease (CVD) [1,2]. Extensive research involving randomized clinical trials demonstrate that intensive control of blood pressure lowers the mortality risk in patients with CKD and HTN [3]. African American (AA) persons have a faster rate of progression and increased mortality for both CKD and HTN. These comorbidities lead to elevated risk of developing CVD and higher mortality [4]. The implementation of robust and prognostic translational research approaches in clinical settings, targeting various pathophysiological diseases like CKD, HTN, and CVD, are key for scalable and cost-effective mechanisms to mitigate the burden on public health and healthcare systems. In this study, we explored how the provision of base-producing fruit and vegetables (cardiorenal protective diet) impacted metabolite profiles and health outcomes in AA individuals with both CKD and HTN.

When comparing races, the prevalence of HTN, treatment eligibility, and unmet treatment goals are significantly higher among AA persons [5]. Individuals who are older, diabetic, male, have higher body weight, and have higher CVD risk are especially vulnerable [5]. In the United States, CKD and ESRD depict one of the most dramatic illustrations of racial and ethnic disparities in health, where AA persons have over three times higher risk for renal replacement therapy than non-Hispanic Whites [6]. Compared to White individuals, AA persons have a higher prevalence of overweight/obesity, diabetes, and HTN. Health beliefs and behaviors as well as social determinants of health (SDoH) (i.e., health literacy, income, insurance, and community resources) are important considerations for understanding existing health disparities [6]. Overall, racial and ethnic inequalities in wellbeing may be correlated with disease progression and severity as well as increased mortality due to biological factors and comorbidities within population groups [7]. 

Racial and ethnic differences that drive health inequalities, such as CKD, ESRD, and CVD, are complex and require a thorough investigation of all confounding factors that may improve the standard practice of clinical care. For example, Wu et al. reported no causal effect between estimated glomerular filtration rate (eGFR) and serum uric acid concentrations in individuals of African ancestry, but a causal effect was found in those of European and Asian ancestries, indicating the importance of racial and ethnic disparities in medical care [8]. Similarly, Reynolds et al. found an ancestry-driven metabolite variation in the *ALMS1* gene associated with CKD in those of African descent [9]. The prevalence of the high-risk alleles G1 and G2 of the *APOL1* gene, which impair mitochondrial function and downstream metabolic pathways, is associated with CKD and ESRD in AA descendants [10]. However, research must transcend beyond studies targeting genomic disparities to understand the social and biological variances that impact CKD in AA individuals. 

Diet, a component of SDoH, plays a considerable role in health outcomes and progression as well as the severity of chronic diseases like CKD. Adults experiencing poverty have less access to health-promoting foods, which increases risks for CKD, HTN, and ESRD development [11,12]. Diet-specific studies and interventions have been investigated in individuals with CKD to evaluate whether dietary intake lowers the risk of CKD progression. A food pattern study by Mazidi et al. suggested that trace elements and vitamin intake are associated with reducing the risk of CKD [13]. Additionally, lower adherence to the Dietary Approaches to Stop Hypertension (DASH) diet—an intervention to lower blood pressure—was associated with greater ESRD risk in AA individuals with moderate CKD and hypertension [14]. Valle-Hita et al. reported the impact of dietary acid load on kidney function decline, where higher renal acid load and net endogenous acid production resulted in ≥10% eGFR decline and ≥10% urine ACR increase [15]. Several studies have supported the impact of diets on CKD progression, where diets heavy in fatty foods and animal-based protein harm kidney health and Mediterranean, fiber-rich, plant-based diets are beneficial due to their alkalizing effects [16,17,18].

Overall, understanding how to improve cardiorenal outcomes in those at higher risk due to SDoH through dietary approaches is understudied. Despite knowing that limited access to healthy diets as well as the socioeconomic and ethnic backgrounds of patients with CKD and HTN are added risk factors for ESRD, the cumulative effects of these risk factors on CVD progression and outcomes remain limited. Considering that racial and ethnic minority communities have disproportionally lower socioeconomic status, options for healthy foods and medical care may be limited; thus, it is critical to identify an effective, scalable, and cost-effective dietary intervention with promising cardiorenal outcomes. Therefore, community-based early detection of kidney function biomarkers indicating CKD status and advancement, supplemented with the treatment of a simple cardiorenal protective diet, may be a practical tool for screening and treatment.

The utilization of metabolomic profiling in the treatment and diagnosis of chronic diseases is a novel and emerging field in science that potentially serves as a powerful tool to improve health outcomes [19,20]. Metabolomics is the comprehensive study of small metabolites and analytes that are derived from metabolic processes occurring within an organism [21,22]. Understanding the interaction between metabolites and their biochemical effects in organisms reveals the subcellular activity of cells and tissues, which impacts metabolism and overall health and wellbeing. With growing knowledge obtained from metabolomic analysis, the early detection of disease and development of precise and personalized therapeutics is highly feasible for improving population health outcomes [23,24].

Metabolites serve as prognostic indicators of disease due to their subcellular molecular and biochemical functions, indicative of pathophysiological behaviors prior to their clinical prognosis or diagnosis. For example, the use of metabolomic analysis has allowed research to refine the hallmarks of CKD that can identify potential biomarkers and causal pathways of disease progression [19]. Determining the distinct functions of metabolites can lead to the development of early diagnostic screening tools and highly specific novel treatments to prevent disease progression. In addition to gaining insights into disease, metabolomic assessment provides robust information on nutrition that can aid in the prevention and management of chronic diseases [25,26,27]. Current findings elucidate the impact of dietary interventions on health using metabolomic profiling to gain a physiological understanding of clinical data. Rubio-Aliaga et al. reported that a 12-week high-fat diet led to significant changes in one-carbon metabolism in the liver, shedding light on how excess fat leads to insulin resistance caused by the alteration of the lipid milieu and increased phosphocholine concentrations [28]. The widespread utilization of metabolomic profiling is rapidly evolving and may soon become a routine practice for disease prevention, management, and treatment.

In the last decade, metabolomics has been widely applied in studies focusing on obtaining a subclinical understanding of the high incidence levels of CKD progression in the AA community [19,29,30,31,32]. Mwasongwe et al. explored the utilization of a multi-marker panel to predict the risk of CKD and rapid kidney function decline (RKFD) in AA adults [33]. The multi-marker panel approach to identify biomarkers for renal outcomes reported a moderate predictive improvement in CKD and RKFD incidence. To date, community-based studies that assess kidney disease biomarkers to stratify disease risk remain limited [33,34,35]. Weekley and Peralta have stated that CKD classification and risk stratification may be improved by utilizing combinations of multiple biomarkers other than the traditionally used albumin–serum creatinine ratio indicator [36]. Furthermore, multi-marker approaches may elucidate the progression and severity of kidney injury by associating specific markers with different pathophysiological pathways, including CVD risk [37].

In this study, a simple cardiorenal protective diet was implemented to evaluate the impact of fruit and vegetables (FVs) in AA individuals with CKD (Stage 1–3) and HTN over a 6-week intervention in a sustainable community care clinic setting. We implemented a base-producing FV intervention to evaluate the impact of a dietary-alkali-promoting condition [38] to better understand cardiorenal outcomes. The primary hypothesis was that FV intake will lead to improvements in health outcomes that prevent or minimize CVD risk. Our secondary hypothesis was that metabolomic profiling will identify how the cardiorenal protective diet (increase in FV intake) affects cardiorenal outcomes, specifically CKD progression and severity towards CVD risk.

## 2. Materials and Methods

### 2.1. Study Participants

This interventional study was approved by the Institutional Review Board of Baylor Scott & White Research Institute (IRB Number: 018-095). Initial eligibility was determined by participants completing a screening form, blood pressure measurements, and a point of care urine dipstick protein analysis. Participant eligibility criteria included the following: 1. men and women ≥ 18 years of age; 2. willingness to participate in a 3-month study; 3. chronic kidney disease (CKD) stage 1–3; 4. diagnosis of hypertension (HTN) and taking medications and/or systolic blood pressure (SBP) > 140 mm/Hg and/or diastolic blood pressure (DBP) > 90 mm/Hg; and 5. African American (AA) race, self-reported. Ineligible individuals included the following: 1. receiving or needing kidney dialysis in the near future (CKD Stage 4–5); 2. meeting recommended levels of blood pressure for individuals with CKD (i.e., SBP < 140 mm/Hg; DBP < 90 mm/Hg (JNC8)) [39] and not taking blood pressure medication; 3. received or needing a kidney transplant, 4. pregnant or planning to become pregnant; and 5. baseline urinary potassium levels > 4.6 mEq/L. This study employed restrictive sampling to AA persons due to the potential differences in biomarkers, metabolites, and disease risk in the focus population [40,41,42,43]. The study population included clinical patients and community members of the Baylor Scott & White Health and Wellness Center (Dallas, TX, USA).

### 2.2. Study Design

**Figure 1:**
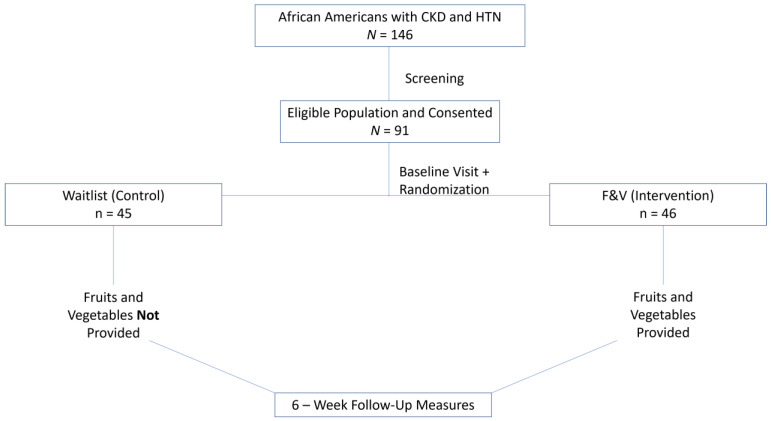
Overall Study Design. The sample population included African American individuals of 18 years or older who were diagnosed with chronic kidney disease and hypertension. Individuals were screened to identify eligibility. Consented participants were measured at baseline and randomized to the control or intervention group. The control group did not receive fruit and vegetables, whereas the intervention group was provided with fruit and vegetables. After 6 weeks, all participants were measured for post-intervention changes.

This study comprised three phases: 1. screening, 2. intervention, and 3. follow-up. Individuals (*N* = 146) were screened using inclusion and exclusion criteria as well as a preliminary point of care urine analysis for urine proteinuria, microalbumin, creatinine, and potassium levels. The “Cardiorenal Protective Diet Trial” was a prospective, 2-arm study that randomized 91 individuals to either a 6-week fruit and vegetables (F&V or FV) community care clinic intervention to improve cardiorenal risk (n = 46) or a waitlist (WL) usual care condition (n = 45) (Figure 1). Participants were measured for physiological data at baseline, 6 weeks post-intervention (~42–56 days), and at 3-month follow-up (~90–110 days).

Participants randomized to the cardiorenal protective FV intervention received the prescribed amount of fresh, base-producing F&V (2 cups daily) cost-free for 6 weeks, followed by 6 weeks of farm stand vouchers. Individuals in the FV group collected the F&V bags from Baylor Scott & White Health and Wellness Center, which was conveniently located in proximity to their homes or workplaces, on a weekly basis. Participants were familiar with the community care center and collection times and days were flexible to lessen participant burden. If the participant missed the pickup, they were reminded via text message or phone call by the research team. The FV group was instructed to increase their intake of F&V to a minimum of 2 cups/day in addition to their routine diet. Whereas the usual care WL control group only received farm stand vouchers for F&V at the end of the study (6 weeks post-intervention). During the 6-week intervention, the WL group continued their routine diet without the provision of F&V. Both the intervention and control groups were also measured at 3-month follow-up for health outcomes (data not included in this paper).

### 2.3. Health Outcomes

Various physiological measures, such as abdominal obesity (waist circumference and body mass index [BMI]), kidney health (albumin to creatinine ratio [ACR]), SBP, and DBP were recorded to evaluate health outcomes. Additional measures, such as glycosylated hemoglobin A1C (HbA1C), high-density lipoprotein (HDL), and total cholesterol (TC), were also measured. Weight was measured with a digital scale and height with a stadiometer. A tape measure was used to measure waist circumference. All body measures were taken twice and averaged. Creatinine and albumin were measured for the ACR using the McKesson Consult^TM^ 120 Urine Analyzer (Irving, TX, USA). Additionally, SBP and DBP were measured with the Omron (Kyoto, Japan) digital blood pressure monitor. Fasting glucose and HbA1C measures were collected using the Siemens DCA Vantage Analyzer (Camberley, UK). Lastly, the lipid panel consisting of TC, HDL, low-density lipoprotein (LDL), and triglycerides (TG) was measured with the Alere Cholestech LDX Analyzer (Waltham, MA, USA).

### 2.4. Metabolomic Analysis

The secondary aim of this study identified circulating biomarkers assessed by metabolomic profiling. The study groups (WL control and FV intervention) consisted of AA adults with CKD and HTN, whereas the metabolic control group comprised of AA adults who had no indication of HTN and CKD (CKD0). The metabolic control group (n = 20) included unrandomized and unblinded healthy participants who were not diagnosed with CKD and HTN previously or had controlled blood pressure and did not receive intervention.

Eligibility requirements for participants in the healthy control group for the metabolomic analysis (metabolomic control) included the following: 1. males and females age ≥ 18 years; 2. willingness to participate in a one-day study; 3. no diagnosis of CKD, self-reported; 4. no diagnosis of HTN or controlled HTN (i.e., SBP < 140 mm/Hg; DBP < 90 mm/Hg with the use of medication) (JNC8) [39], self-reported; and 5. AA race, self-reported. Exclusion criteria for the metabolomic control group were the following: 1. not of AA race; 2. CKD, all stages; 3. diagnosis of HTN and/or SBP ≥ 140 mm/Hg and/or DBP ≥ 90 mm/Hg; 4. received or needing a kidney transplant; 5. received or needing dialysis; and 6. pregnant.

Blood was collected for the metabolomic analysis at baseline and 6 weeks post-intervention timepoints. Blood was collected for the metabolic control group at a one-day timepoint during the study. Plasma was collected utilizing a dried plasma spot (DPS) collection card (NoviplexTM, Novilytic Labs, West Lafayette, IN, USA), which is a miniaturized blood fractionation technology enabling the collection of plasma from a few drops of blood (~3 µL) obtained by a finger stick. Two plasma spots were collected from each subject and immediately stored at −80 °C until the time of analysis. Quantitative targeted metabolomic analysis was performed using the MxP^TM^ Quant 500 kit (Biocrates Life Sciences Ag, Innsbruck, Austria) following the manufacture’s protocol for a 5500 QTrap instrument (Sciex, Framingham, MA, USA) [44]. Typically, the method requires 10 µL of plasma for optimal performance. However, we adapted the method to use two plasma spots (corresponding to 6 µL of plasma) that were placed directly into the wells of the kit plate. The final results for the concentration of metabolites were corrected for the lower volume of plasma used.

Metabolites were measured by liquid chromatography–mass spectrometry (LC-MS) and flow injection analysis–tandem mass spectrometry (FIA-MS) instrumentation. The MxP^TM^ Quant 500 kit has a coverage of up to 630 metabolites from 25 biochemical classes with a wide range of analytes. Peak alignment, filtering, and normalization were performed using METIDQ (Biocrates Life Sciences Ag, Innsbruck, Austria). Further analysis was performed using MetaboINDICATOR^TM^ (Biocrates Life Sciences Ag, Innsbruck, Austria), which calculates up to 232 metabolic indicators from the ratio and/or sums of metabolites. These metabolomic indicators can provide useful insight into biochemical pathways.

### 2.5. Statistical Analysis

#### 2.5.1. Health Outcomes and FV Intake

Group comparisons between baseline and 6 weeks post-intervention for health outcomes and FV intake were performed via two sample *t*-tests. A general linear model was used to evaluate the group effects of the FV intervention from baseline to 6 weeks. Statistical significance was determined using a *p* value of 0.05.

#### 2.5.2. Metabolomic Analysis

Statistical analysis was conducted to determine which analytes were significantly overexpressed and underexpressed for CKD (Stage 1–3). Data preprocessing included exporting longitudinal Quant500 metabolites from MetIDQ and conducting the QC process. PBS negative controls were used to determine the lower limit of detection (LOD) to filter metabolites, where 500 metabolites passed filtering. Then, QC2 positive controls were utilized to align metabolite concentrations between the two plates of the study. Metabolomic indicators were calculated based on existing scripts on the available metabolites passing filtering, which resulted in 185 indicators. Each metabolite/indicator concentration was converted to normal distribution via Box–Cox transformation and then standardized to the “waitlist control” group for downstream statistical analyses (R 3.3.2 Statistical Program).

For data visualization, unsupervised principal component analysis and hierarchical clustering were utilized to visualize the pattern of all samples. Differential analysis was performed via a linear mixed model to account for the repeated measurements, with the main factors of time point, treatment arm, CKD group, as well as covariates of age, gender, BMI, and clinical conditions of diabetes, HTN, obesity, and cholesterol. Comparisons were performed on contrasts, such as all study participants vs. metabolic CKD0 control, post-intervention vs. baseline, CKD2/3 vs. CKD1, and among others, for each treatment arm separately or in a joint fashion. To graphically represent results based on the linear mixed model, forest plots were utilized to illustrate the expression pattern of a panel of significant metabolites/indicators, such as between two time points or across the CKD groups for each treatment arm.

## 3. Results

### 3.1. Demographic Data

Table 1 shows baseline demographic data including age, sex, total household income, education level, marital status, and health outcomes collected from all study participants (*N* = 91). Forty-five participants were randomized to the WL group, and forty-six participants were randomized to the FV group. The average (standard deviation [SD]) age of the participants was 58.3 (10.1) years, 66% were female, and 100% were self-identified as Black/African American. Approximately 65% of the participants had an annual household income of less than USD 50,000, 32% had a high school degree or less, and 30% were married.

### 3.2. Fruit and Vegetable Intake

Adherence to the Cardiorenal Protective Diet was monitored by recording weekly pickups of base-producing F&Vs, which were pre-bagged for participants. Throughout the 6-week intervention, participants obtained 5.5 out of 6 pickups, resulting in 91.7% adherence. The change in F&V consumption among the groups was assessed through self-reported data collected via 2-item FV serving screening questions (adapted from the Fruit & Vegetable Intake Screeners in the Eating at America’s Table Study [45]) at baseline and 6 weeks post-intervention. The questions were asked in the context of usual eating or drinking habits. Short screening questions were utilized to minimize participant burden. Both groups had an overall nonsignificant increase in F&V intake from baseline to 6 weeks (FV group: 0.6 increase in fruit servings and 0.5 increase in vegetable servings; WL group: 0.4 increase in fruit servings and 0.4 increase in vegetable servings).

### 3.3. Health Outcomes

Change scores (6 weeks—baseline) for health outcomes by study groups are displayed in Table 2. The change scores for FV and WL represent measured health variables to determine the effectiveness of adding a minimum of 2 cups of F&Vs per day to a routine diet in AA individuals diagnosed with CKD and HTN.

Change scores for health outcomes from baseline to 6 weeks post-intervention were not statistically significant (Table 2). The change score for urine ACR was −5.5 ± 19.9 mg/g for the FV group and 1.8 ± 24.8 mg/g for the WL group (*p* = 0.21), whereas BMI and SBP had nonsignificant reductions in both the FV and WL groups. TC had a change score of −1.3 ± 23.8 mg/dL for the FV group and 0.30 ± 22.4 mg/dL for the WL group (*p* = 0.78).

### 3.4. Metabolomic Analysis

#### 3.4.1. CKD Effects for All Study Participants at Baseline

**Figure 2:**
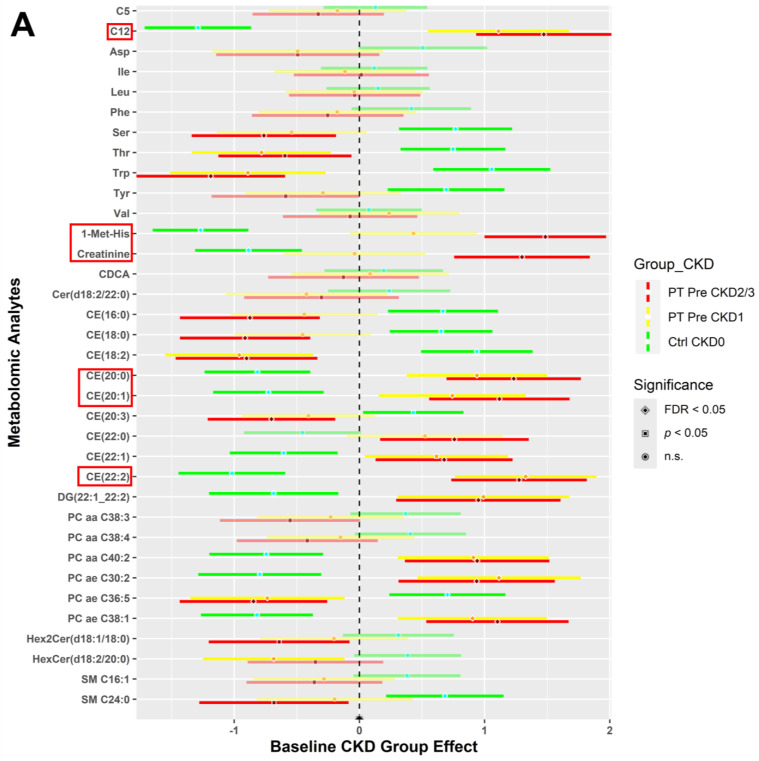

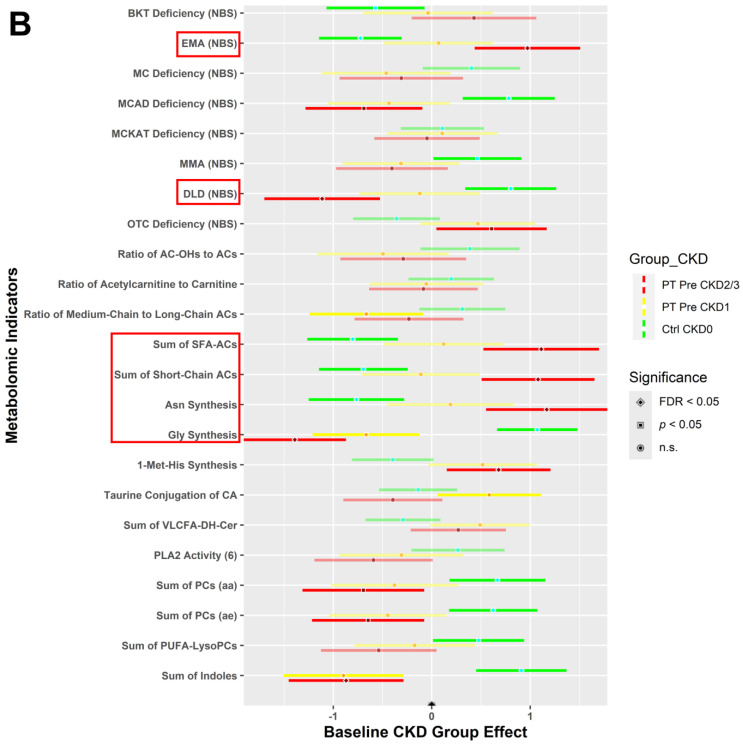
All study participants (WL control and FV intervention) vs. metabolic control (CKD0) individuals at baseline. Analytes from blood samples were collectively analyzed for all participants irrespective of the randomization group at baseline. A two sample *t*-test and chi-squared test with FDR correction, comparing each CKD stage (1 and 2/3) to CKD0, were performed for statistical significance (*α* = 0.05) to determine expression levels. These forest plots indicate the pre-intervention expression levels of metabolites (**A**) and indicators (**B**) with respect to the CKD stages 0 (green), 1 (yellow), and 2/3 (red). Significant metabolites and indicators of interest (dark shaded colored bars) are highlighted with a red box.

Plasma from blood samples collected for all study participants combined into one group at baseline were analyzed. Figure 2 displays the expression of metabolomic analytes (A) and metabolic indicators (B) in forest plots by CKD group effect. Data for both plots were separated by CKD stages (0, 1, and 2/3) to distinctively model varying expression levels of metabolites and indicators pre-intervention. The forest plot also highlights the levels of expression of other metabolites and indicators by CKD stages for comparison; however, they are not statistically significant (lightly shaded bars with circles). Significant metabolites and indicators (dark shaded colored bars) of interest are highlighted with a red box.

The expression of C12 (dodecanoylcarnitine) and cholesteryl esters: CE (20:0), CE (20:1), and CE (22:2) were significantly higher (FDR < 0.05) for participants with CKD stages 1 and 2/3, as compared to CKD0 (Figure 2A). 1-Met-His (1-methylhistidine) and creatinine metabolite expression were highest for CKD2/3, intermediate for CKD1, and lowest for CKD0. Other metabolites had the opposite group effect, where these metabolites were expressed higher for the CKD0 group but lower for CKD1 and CKD2/3: amino acids—serine (Ser), threonine (Thr), tryptophan (Trp); cholesteryl esters—CE (16:0), CE (18:0), CE (18:2), and CE (20:3); acyl-alkyl-phosphatidylcholine—PC ae C36:5; dihexosylceramide—Hex2Cer (d18:1/18:0); and sphingomyelin—SM C24:0.

Figure 2B portrays the expression levels of selected indicators for groups CKD0, CKD1, and CKD2/3 for all study participants at baseline. Indicators such as ethylmalonic encephalopathy—EMA (NBS), dihydrolipoamide dehydrogenase deficiency—DLD (NBS), sum of saturated fatty acid acylcarnitines (SFA-ACs), sum of short-chain acylcarnitines (ACs), asparagine (Asn) synthesis, and glycine (Gly) synthesis had a gradient effect between the CKD groups. However, CKD1 and CKD2/3 have a similar expression pattern compared to CKD0 for other indicators such as medium-chain acyl-coenzyme A dehydrogenase (MCAD) deficiency (NBS), ornithine transcarbamylase (OTC) deficiency (NBS), 1-Met-His synthesis, sum of diacyl-phosphatidylcholines (PCs (aa)), sum of acyl-alkyl-phosphatidylcholines (PCs (ae)), and sum of indoles.

#### 3.4.2. CKD within Group Effects at Baseline vs. 6 Weeks Post-Intervention

**Figure 3:**
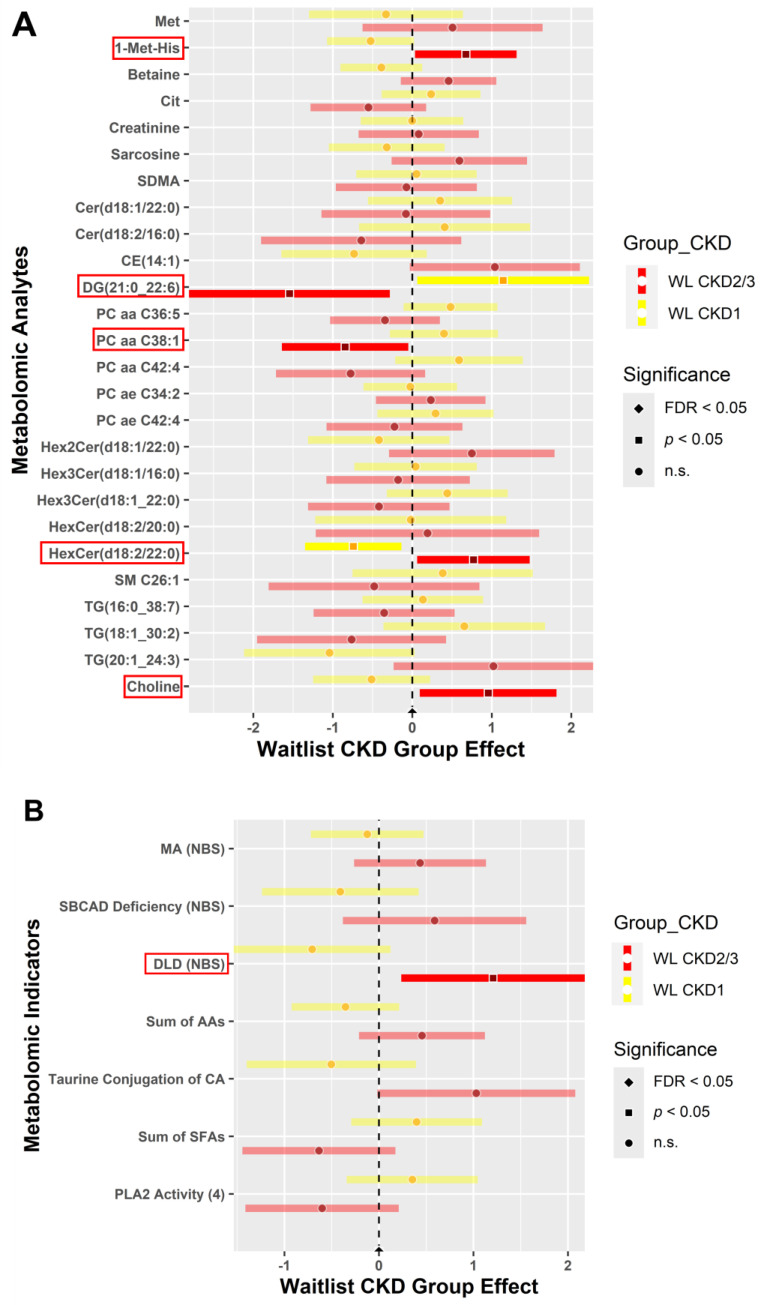
Metabolites (**A**) and indicators (**B**) comparing expression between baseline and post-intervention for the waitlist (control) group. Blood samples collected at baseline and 6 weeks post-intervention were analyzed for the waitlist (control) group. Expression levels of metabolites (**A**) and indicators (**B**) in CKD stages 1 (yellow) and 2/3 (red) are depicted by forest plots. A two sample *t*-test and chi-squared test with FDR correction, comparing each CKD stage, were performed for statistical significance (*α* = 0.05) to determine expression levels. Significant metabolites and indicators (dark shaded colored bars) of interest are highlighted with a red box.

**Figure 4:**
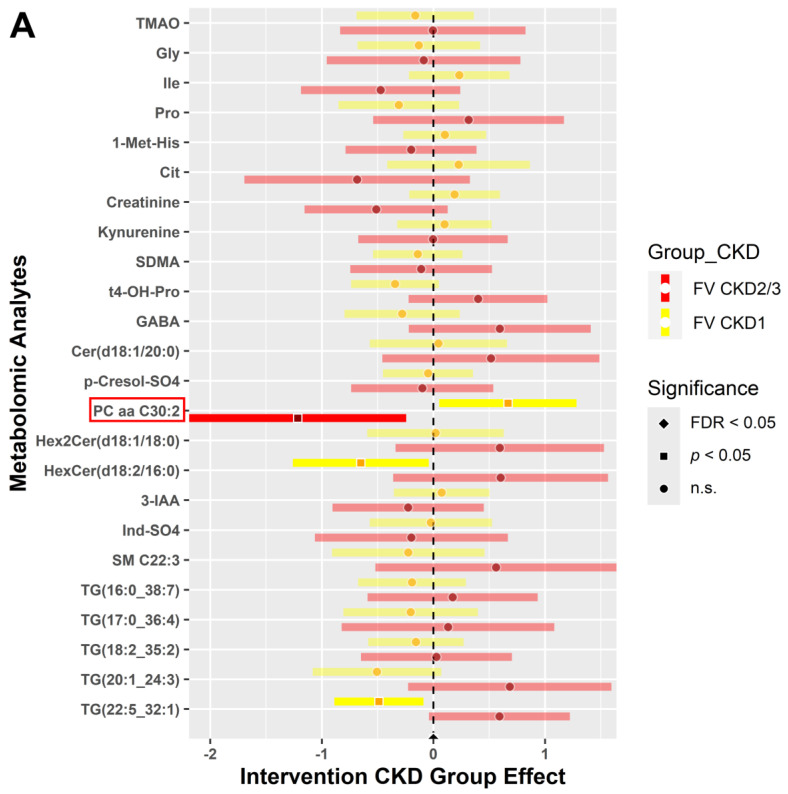

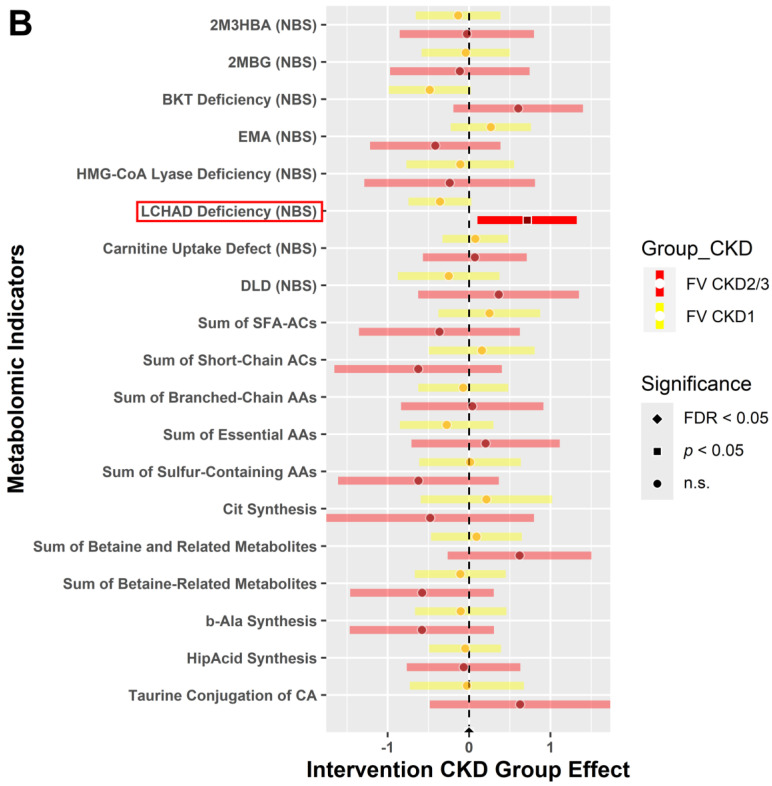
Metabolites (**A**) and indicators (**B**) comparing expression between baseline and 6 weeks post for the intervention (FV) group. Blood samples collected at baseline and 6 weeks post-intervention were analyzed for the FV (intervention) group. Expression levels of metabolites (**A**) and indicators (**B**) in CKD stages 1 (yellow) and 2/3 (red) are displayed by forest plots. Two sample *t*-test and chi-squared test with FDR correction, comparing each CKD stage, were performed for statistical significance (*α* = 0.05) to determine expression levels. Significant metabolites and indicators (dark shaded colored bars) of interest are highlighted with a red box.

A comparison of 6 weeks post-intervention and baseline results showed changes in metabolite and indicator expression levels (post- minus pre-intervention) for the WL (Figure 3) and FV (Figure 4) groups separately. Forest plots representing the changes in metabolite (A) and indicator (B) expression levels between baseline and post-intervention for participants randomized to the WL group are shown in Figure 3. The WL-CKD2/3 group demonstrated higher expression of metabolites 1-Met-His, hexosylceramide—HexCer (d18:2/22:0), and choline, but lower levels of diglyceride—DG (21:0_22:6) and phosphatidylcholine—PC aa C38:1 than the WL-CKD1 group (*p* < 0.05) (Figure 3A). In addition, the WL-CKD2/3 group demonstrated higher levels (*p* < 0.05) of the DLD (NBS) indicator (Figure 3B). Significant metabolites and indicators (dark shaded colored bars) of interest are highlighted with a red box.

Furthermore, forest plots illustrating the changes in metabolite (A) and indicator (B) expression levels between baseline and post-intervention for participants randomized to the FV group are shown in Figure 4. Intervention participants displayed fewer significant metabolites compared to the WL participants in Figure 3. In the comparison of pre- and post-intervention, the FV group portrayed lower expression levels of phosphatidylcholine—PC aa C30:2 (*p* < 0.05) in participants with CKD2/3 (Figure 4A). Additionally, Figure 4B shows that the indicator for long-chain 3-hydroxyacyl-coenzyme A dehydrogenase (LCHAD) deficiency (NBS) was higher in FV participants with CKD2/3 (*p* < 0.05). As mentioned before, the lightly shaded bars with circles indicate nonsignificant levels of expression. Significant metabolites and indicators (dark shaded colored bars) of interest are highlighted with a red box.

#### 3.4.3. CKD between Group Effects at Baseline vs. Post-Intervention

**Figure 5:**
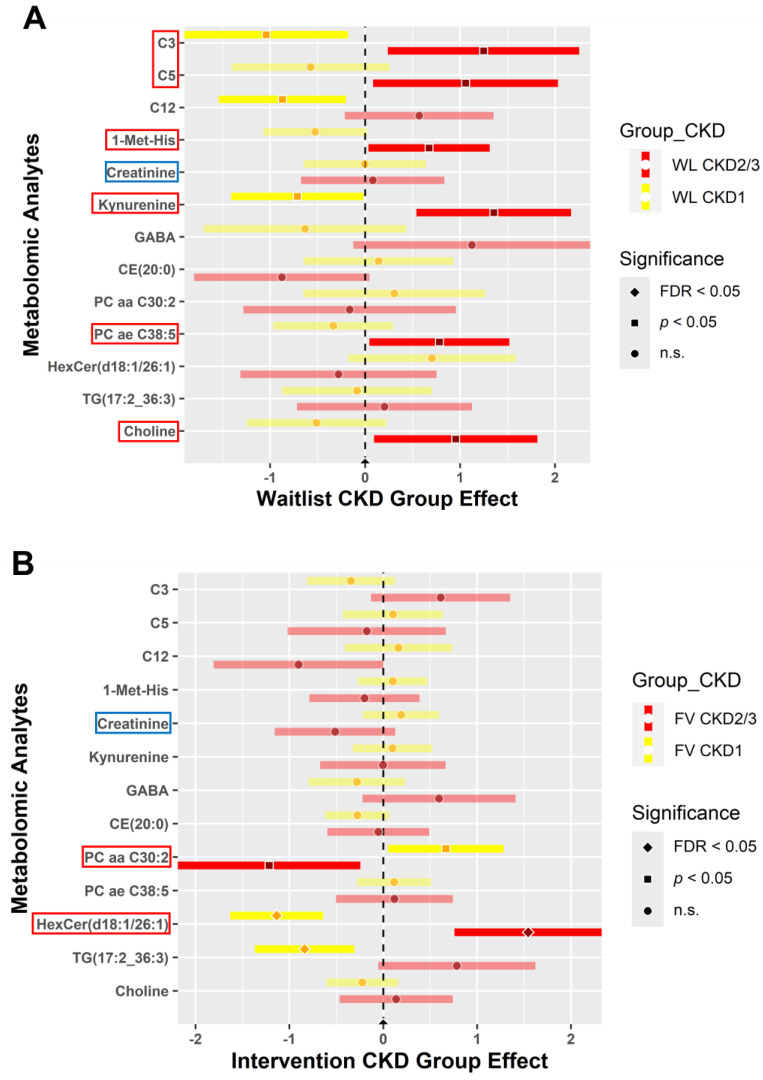

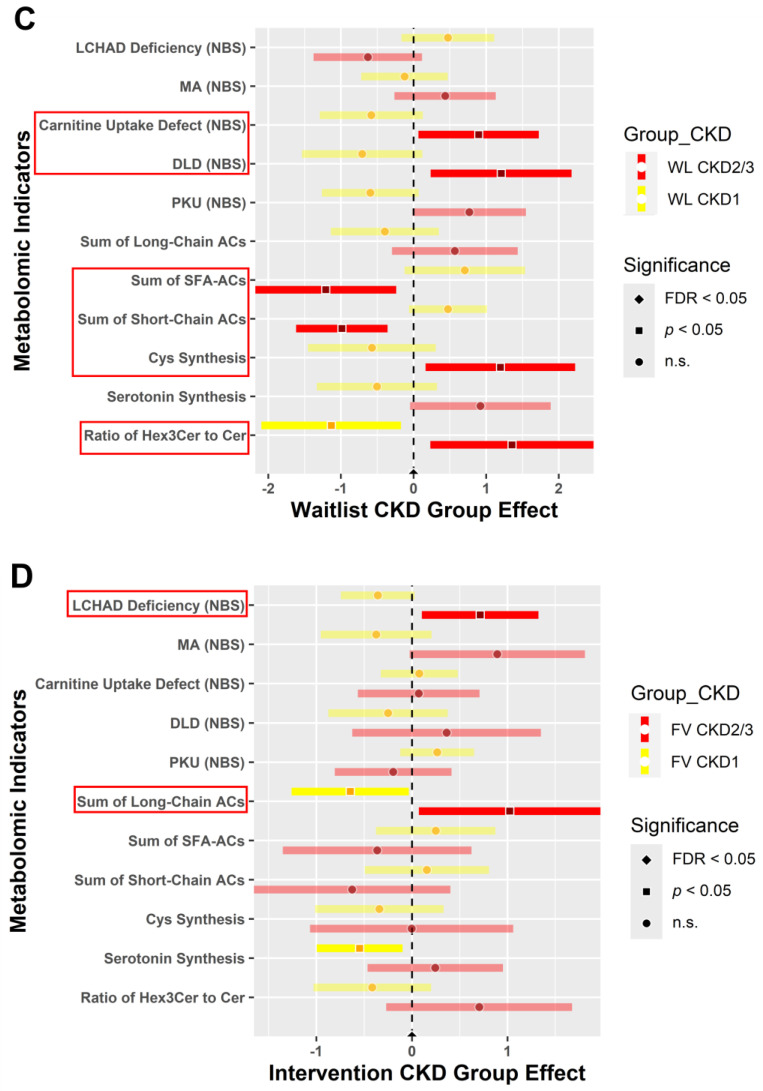
Metabolites and indicators for the waitlist (control) and FV (intervention) groups between baseline and 6 weeks post-intervention. Blood samples collected at baseline and 6 weeks post-intervention were analyzed for the waitlist (**A**,**C**) and FV intervention (**B**,**D**) groups. Expression levels of metabolites (**A**,**B**) and indicators (**C**,**D**) in CKD stages 1 (yellow) and 2/3 (red) are represented by forest plots. The red boxes highlight significant metabolites and indicators, whereas the blue box highlights interesting findings for creatinine analyte expression. These plots depict post- minus pre-intervention changes over time for the metabolomic analytes and indicators between the study groups. Two sample *t*-test and chi-squared test with FDR correction, comparing each CKD stage, were performed for statistical significance (*α* = 0.05) to determine expression levels.

The forest plots in Figure 5 show the changes (post- minus pre-intervention) in expression levels of metabolomic analytes and indicators between the WL and FV groups, representing a group as well as time effect. We selected identical significant metabolites and indicators for a closer comparison of variables for the WL group (Figure 5A,C) and FV group (Figure 5B,D). The names of significantly expressed metabolomic analytes and indicators (dark shaded colored bars) of interest are highlighted with a red box. The blue box highlights nonsignificant, yet interesting, findings for creatinine (Figure 5A,B).

The WL group demonstrated higher levels of metabolite expression in participants with CKD2/3 for C3 (propionylcarnitine), C5 (valerylcarnitine), 1-Met-His, kynurenine, PC ae C38:5, and choline (*p* < 0.05) (Figure 5A). On the other hand, when compared to CKD1, PC aa C30:2 had reduced expression in CKD2/3 (*p* < 0.05) yet increased expression of HexCer (d18:1/26:1) in CKD2/3 (FDR < 0.05) FV participants (Figure 5B). Creatinine (blue box) is highlighted in the forest plots for the WL and FV groups (Figure 5A,B). Although creatinine expression was not statistically significant, it reduced in participants with CKD2/3 when compared to CKD1 individuals in the FV group (Figure 5B). The WL participants with CKD2/3 and CKD1 did not show changes in creatinine expression levels (Figure 5A).

In the WL group, metabolomic indicators revealed higher levels of carnitine uptake defect (NBS), DLD (NBS), Cys (cysteine) synthesis, and the ratio of Hex3Cer to Cer (ratio of trihexosylceramides to ceramides) (*p* < 0.05), yet decreased levels of the sum of SFA-ACs and sum of short-chain ACs for CKD2/3 when compared to CKD1 (*p* < 0.05) (Figure 5C). However, only two recognized metabolomic indicators appeared significant in the FV group. Indicators for LCHAD deficiency (NBS) and the sum of long-chain ACs were higher in CKD2/3 participants than individuals with CKD1 in the FV group (*p* < 0.05) (Figure 5D).

## 4. Discussion

Chronic diseases, such as HTN, diabetes, elevated cholesterol, CKD, and obesity [46,47,48,49], can lead to increased CVD risk, especially for AA persons [50]. This randomized parallel two-arm trial demonstrated the feasibility of a FV intervention for community-dwelling AA individuals diagnosed with CKD and HTN. In the current study, we incorporated a base-producing F&V intervention to reduce metabolic acidosis and evaluate the impact of a dietary-alkali-promoting condition in individuals with CKD [38] to understand its impact on cardiorenal outcomes. The provision of F&V is a cost-effective, feasible, and scalable community-based intervention to improve health outcomes. This feasibility trial incorporated a unique study design, including measures of clinical health as well as metabolomic profiling, to identify subclinical and biological changes over time in a community care setting.

We measured selected health variables (SBP, DBP, HDL, LDL, TC, TG, BMI, HbA1C, and ACR) which implicate CVD risk in this study. Within a 6-week intervention period, we found nonsignificant outcomes in measured variables. Previous studies reported that a base-producing FV intervention was correlated with significant ACR reductions at 4 weeks [51]; however, our findings showed nonsignificant ACR reduction at 6 weeks, supporting a similar FV intervention study [52]. Unlike earlier studies that provided 2 cups of F&V per day to the entire participant household, the present feasibility trial provided F&V only to study participants [38,51,53,54]. Additionally, another FV intervention trial provided food preparation instructions adjunctive to F&V and the intervention group experienced ACR reduction [52]. By providing cooking instructions [52] or guided goal setting with the participant [55,56], the FV intervention improved dietary and observed health outcomes. Considering the cultural, social, dietary, and lifestyle variations among AA communities, FV interventions may need multi-theoretical approaches (i.e., targeting community, interpersonal, or individual levels [socioecological model [57]]) for optimal clinical outcomes.

Overall, the consumption of the basic 2 cups of F&V per day positively impacted health variables with nonsignificant reductions in ACR, BMI, TC, and SBP in this study, indicating that a prolonged incorporation of the minimum dosage of F&V into a routine diet may lead to other significant health outcomes, CKD management, and decreased cardiorenal risk. Meslier et al. demonstrated that a Mediterranean diet intervention, which included increased intake of F&V, in overweight and obese individuals lowered plasma cholesterol [58]. Additionally, F&V reduce phosphorus burden, have favorable effects on potassium metabolism, and contain nutrients that are anti-inflammatory and antioxidative [59], mitigating negative physiological effects on renal and cardiovascular function. Hyperlipidemia or hypercholesterolemia, overweight or obese condition, and elevated ACR are associated with CKD and CVD risk [1,47,60]. CKD, which is correlated with dyslipidemia, results in the downregulation of lipoprotein lipase and the LDL-receptor as well as delayed metabolism and the clearance of triglyceride-rich lipoprotein and lipids, elevating lipid (cholesterol, LDL, and TG) levels [61]. Findings report that patients with nephrotic syndrome have reduced LDL-receptor activity but increased acyl-CoA cholesterol acyltransferase (ACAT) and HMG-CoA reductase activity, which results in higher LDL-cholesterol levels [61]. F&V provides low fat, dietary fiber (reduces the absorption of fats and cholesterol), phytosterols (inhibits the intestinal absorption of cholesterol, cholesterol displacement from mixed cellular micelles, sterol uptake interference, and cholesterol excretion stimulation), micronutrients (vitamins and minerals), and phytochemicals (i.e., polyphenols, carotenoids, anthocyanins, and quercetin) [62], which provide anti-inflammatory, antioxidant, and gut health-promoting properties to reduce hypercholesterolemia [62]. Similarly, F&V have protective characteristics, such as natural phenols, terpenes, flavonoids [63], anthocyanin (glucose transporter 4 regulation), and quercetin (protection against β-cell damage and oxidative stress reduction) [64], enhancing kidney function. 

We demonstrated that metabolomic analysis can improve our understanding of subclinical changes as significant improvements in health outcomes may require a longer duration of time or large doses of F&V. The indicators in this study highlight metabolic dysfunction, which is common to those with CKD. The upregulated expression of the carnitine uptake defect indicator in the WL group for CKD2/3 can be attributed to the inefficient β-oxidation of fatty acids. Carnitine is necessary to transport acyl-CoAs and fatty acids into the mitochondria using carnitine–acylcarnitine translocase [65]. Defective carnitine uptake can hamper the transport processes leading to the buildup of acylcarnitine. Similarly, the upregulation of the LCHAD deficiency indicator in the FV-CKD2/3 group may be characterized by defective mitochondrial fatty acid β-oxidation [66]. Although LCHAD deficiency is a rare fatty acid oxidation disorder in infants, limited studies in adults report disrupted metabolic function leading to severe cardiac consequences like cardiomyopathy and cardiac arrest [67], which may be due to toxin accumulation from kidney dysfunction over time [66]. Additionally, elevated levels of the DLD (NBS) in the WL-CKD2/3 group may be a result of metabolic abnormalities like persistent acidosis [68]; however, this indicator remains understudied. Cysteine synthesis is upregulated in advanced CKD stages in the WL group, which can lead to toxic subcellular consequences, such as autooxidation, protein misfolding, the overstimulation of other metabolic pathways, or endoplasmic reticulum stress, which is continuously driven by kidney impairment [69]. In the FV-CKD2/3 group, the sum of long-chain ACs are elevated, perhaps due to mitochondrial dysfunction and continuous oxidative stress leading to alterations in acylcarnitine homeostasis, indicating a biomarker for uremic cardiovascular risk [70]. For the remaining indicators (sum of SFA-ACs, sum of short-chain ACs, and ratio of Hex3Cer to Cer), research related to CKD and CVD remains limited.

Previous studies report that kidney decline and fat disruption work interdependently [71]. Similar to our findings, Bassi et al. conducted metabolomic profiling for individuals with failing kidneys and found a dose–response association between GFR and short-chain acylcarnitine (C4, C12) [72]. As kidney function declined, serum acylcarnitine accumulated. Acylcarnitine accumulates as a result of suboptimal kidney excretion caused by the loss of renal parenchyma in the progression of CKD [72]. The renal parenchyma is the functional filtration component of the kidneys [72,73]. In our study, after intervention with F&V, the significant difference in acylcarnitine between mild and severe CKD was attenuated. PC ae C30:2, a phosphatidylcholine, indicated lower expression in those with more advanced CKD after the FV intervention, whereas PC ae C38:5 had increased expression in the control group in our study, indicating renal cell growth, since the PC level increases when stimulated by growth signals [74]. Since more renal damage is present in the advanced stage, the degree of increase in phosphatidylcholine is predicted to be higher when treatment of F&V is not administered as a compensatory and inflammatory mechanism. 

Next, hexosylceramide (d18:1/26:1) was another potential metabolite indicating the progression of kidney disease. Hernandez-Corbacho et al. studied the effects of aging kidneys using rat models [75]. They found that hexosylceramide levels were the most upregulated ceramides, which were 8–12-fold higher in most aged kidneys [75]. In another study, CKD-induced mice observed hexosylceramide accumulation, which could cause the CKD-associated renal activation of mTOR downstream signaling, tubular injury, endoplasmic reticulum stress, fibrosis, inflammation, impaired kidney mitochondrial function, and oxidative stress [76]. In our study, hexosylceramide was found at significantly higher levels in advanced CKD stages for the FV group. This may be rational because a diseased and aged kidney has similar suboptimal components, such as pro-inflammatory enzymes and cytokines, that inhibit function [75]. Interestingly, in the WL group, no significant effect was found for this metabolite. This may be explained by the disproportionate randomization of individuals with significant irreversible renal injury in the FV group. Based on previous research and our study, understanding the intricacies of fat dysregulation can provide a deeper understanding of kidney structure, function, and pathologies. 

In addition to fat disorder, unfavorable nutrient uptake was apparent in our study results as well as precursors of toxic metabolites. In the WL group, choline had a significant group effect, which has been widely supported by extensive research. Choline expression shows a high association with CKD and is often present in animal-protein-rich diets [77]. Increased concentrations of choline lead to higher trimethylamine N-oxide (TMAO) levels, which is produced when choline is oxidized by the enzyme flavin-containing monooxygenase 3 (FMO3) [78]. TMAO has become a metabolite associated with increased mortality in those with kidney decline [79]. Elevated levels of TMAOs are inversely related to eGFR, a significant feature of kidney disease [76], and associated with renal fibrosis [77]. Furthermore, high TMAO levels increase the severity of adverse cardiac events [80]. Research suggests that TMAO has multiple mechanisms for inducing hypertension, including the upregulation of the PERK pathway, which increases inflammation, and amplifying scavenger receptors causing atherosclerosis [81].

In our study, kynurenine, a protein-bound uremic toxin derived from tryptophan metabolism, which is heightened in inflammatory conditions [82], was upregulated in those with advanced CKD in the WL group. Experimental studies report that kynurenine causes oxidative stress, inflammation, abnormal angiogenesis, vascular thrombosis, and endothelial cell dysfunction through direct stimulation of the aryl hydrocarbon receptor (AHR), leading to suppression of the Wnt activity driven by the β-catenin pathway [82]. Similarly, Mor et al. found that the kynurenine pathway and its metabolites are upregulated in patients with CKD which causes secondary hypertension, but the literature remains unclear as some studies find that kynurenine reduces blood pressure [83].

1-methylhistadine (1-Met-His) was elevated in the WL-CKD2/3 group when compared to WL-CKD1; however, the expression was nonsignificant in both CKD groups of the FV intervention. In an elder Chinese cohort, Liu et al. identified 1-Met-His as one of the six metabolites that was a predictor for CKD progression due to its abundance in patients with renal dysfunction [84]. Supportive findings from the Baltimore Longitudinal Study of Aging by Yamaguchi et al. found elevated plasma 1-Met-His in adults with CKD when compared to adults without CKD [85]. Similarly, Razavi et al. reported the association of serum 1-Met-His with higher SBP and DBP in AA individuals, but not their White counterparts, suggesting that identifying dietary biomarkers like 1-Met-His may inform racial disparities [86]. These observations may be due to higher meat intake, which is a byproduct of dipeptide anserine metabolism [87], and lower consumption of FV in WL participants. In advanced CKD stages of hypoxia-ischemia renal injury, anserine—a carnosine analog—may produce higher levels of 1-Met-His due to oxidative and nitrosative stress as a counter protective mechanism [88].

Serum creatinine has been a long-standing primary biomarker for screening and diagnosing CKD. However, in our study, it was not significantly different in those with early-stage CKD compared to those with intermediate CKD stages across both WL and FV groups. While it is a standard metric in understanding kidney ailments, it is not always the most reliable. Gounden et al. reported that changes in serum creatinine typically happen during later stages of kidney decline not allowing for early detection and treatment [89]. This may explain the lack of significant differences in creatinine across both groups which include participants with early and mild CKD. Delanaye et al. also undermined the reliability of creatinine in kidney disease, noting how its values vary based on muscle mass, age, ethnicity and gender [90]. The shortcomings of creatinine highlight the need for the expansion of metabolomics in the diagnosis of CKD. With further research, additional molecular markers can supplement serum creatinine to improve early detection methods for a more comprehensive prognosis of kidney disease. 

Collectively, we identified a panel of metabolic marker candidates for predicting CKD progression and CVD risk. Our preliminary data demonstrate a cardiorenal protective mechanism for AA individuals when F&V intake is increased in the daily diet. Based on our findings, we specifically highlight the following metabolites as markers for CKD status and CVD risk: C3, C5, 1-Met-His, kynurenine, PC ae C38:5, choline, and HexCer (d18:1/26:1). Metabolomic indicators that may also serve as screening markers are carnitine uptake defect, DLD (NBS), cysteine synthesis, LCHAD deficiency, and the sum of long-chain ACs. These molecular markers indicate pathophysiological conditions of oxidative stress and inflammation, leading to the disruption of lipid and amino acid metabolism, presence of uremic toxins, renal injury, and cardiovascular stress, which further exacerbates the chronic disease cascade.

As we have shown, metabolomic profiling provides a valuable understanding of the plausible molecular and pathophysiological changes as well as the impact of the provision of F&V in AA persons with cardiorenal risk factors. With increased intake of F&V, C3, C5, 1-Met-His, kynurenine, PC ae 38:5, and choline showed reductions in comparison to the control conditions. Overall, the consumption of FV demonstrates promising molecular and physiological changes. For example, metabolomics may explain the nonsignificant minor reduction in ACR, BMI, TC, and SBP due to reduced fat/lipid absorption, improved excretion, the activation of kidney recovery mechanisms to absorb protein, and anti-oxidative and anti-inflammatory conditions promoted by increased F&V intake. Significant changes in health outcomes take time; however, metabolomic profiling provides a subclinical explanation for intervention outcomes that may enhance targeted therapeutic development.

Our study demonstrates that precision nutrition is a promising method for the management and prevention of CKD as well as other underlying metabolic comorbidities [91,92]. Taking into account one’s genetics, food behavior, dietary habits, physical activity, gut microbiomics, and metabolomics, the provision of food could be personalized and tailored to target nutritional recommendations required to address chronic diseases [93]. Further metabolomic studies involving AA individuals to understand the impact of increased F&V intake on cardiorenal risk factors show strong potential in identifying novel biomarkers that can clinically inform specific dietary guidance [94]. Implementing our findings in optimized FV interventions (i.e., with cooking instructions, family-based setting, ASA-24 survey recall) in a larger sample, including other populations with elevated CKD risk such as AA and Hispanic persons, has potential to develop an affordable and accessible precision nutrition framework in the future [94]. Promotion of plant-based diets, starting from clinical recommendations [95] and extending to educational population health approaches, shows promising impacts for both prevention and management of CKD and CVD risk [96].

In the future, implementation of rigorous dietary assessments (i.e., ASA-24 dietary recall survey) would be beneficial to monitor F&V intake. Vázquez-Manjarrez et al. have identified candidate biomarkers of food intake (BFIs) through a systemic review to evaluate tropical fruit consumption [97]. However, the group found a lack in validation and reliability of findings for most tropical fruits except for banana, watermelon, and avocado, which still require further population and dose–response studies [97]. Another extensive review by Brouwer-Brolsma et al. investigated BFIs for leafy, bulb, and stem vegetables, finding that BFIs for vegetables have been understudied and there are no specific candidates identified yet [98]. Therefore, it is critical to continue investigating promising BFIs for F&V through metabolomic profiling as an objective tool to assess consumption of F&V, dietary changes, and intervention adherence.

Furthermore, metabolomics serves as a powerful tool in CKD research due to its potential impact on the early prognostic screening of CKD progression, CKD management strategies, and providing targeted therapeutic avenues for CKD and CVD treatment. The identification of key metabolite and indicator markers through metabolomics may serve as a high-throughput screening tool, where a clinician can make earlier informed decisions on medical treatments for a CKD patient, especially for those in advanced stages. With a non-invasive and robust technique like metabolomic profiling, the immediate need for kidney replacement therapy could be predicted prior to receiving additional standard functional renal test results. Clinical measures (i.e., ACR, lipids, HbA1C) may take months to show significant damages that have already occurred leading to a delayed diagnosis. Furthermore, metabolomics may lead to targeted therapeutic development via the inhibition of accumulated toxins, such as kynurenine, choline, acylcarnitine, and phosphatidylcholines, found in CKD patients. This can promote disease management, especially in high-risk populations that require more than diet and lifestyle modifications.

One limitation of our study is the small sample size (*N* = 91), although it was determined with a power calculation for ACR. The short duration of the intervention (6 weeks) could have limited the ability to observe significant changes in metabolite expression and clinical health outcomes. Dietary intake was measured through a self-reported brief survey collected at baseline and 6 weeks post-intervention, which may have led to biasness if there were errors in self-reporting (i.e., recall errors, social desirability bias, difficulty assessing portion size). Lastly, limited studies on metabolomic profiling of AA persons, specifically those diagnosed with both CKD and HTN, and cardiorenal protective diet implications on biomolecular physiology restricted elaborate comparative analyses. 

## 5. Conclusions

Together, targeted metabolomic analyte and indicator datasets provided complementary biophysiological significance to the measured health outcomes, strengthening the association between the provision of F&V and cardiorenal protective outcomes. In our study, we found that an increase in F&V intake showed small nonsignificant reductions in ACR, BMI, TC, and SBP, as well as significant expression of key metabolites and indicators associated with renal and cardiovascular damage, disrupted lipid and amino acid metabolism, abnormal uptake of lipids and cholesterol, inflammation, and oxidative stress. Therefore, our pilot study provides support for future dietary intervention studies that are cost-effective, sustainable, scalable, and feasible to prevent CKD progression and improve cardiorenal outcomes. The development of a diagnostic and prognostic renal panel identifying specific metabolites and indicator pathways, which lead to the deterioration of kidney function and increase the risk of cardiovascular implications, may lead to substantive therapeutic targets. Overall, our study demonstrates that the early detection of CKD stage, CVD risk, and disease severity using metabolomic profiling can potentially halt CKD progression towards ESRD or CVD, providing a better quality of life to predisposed high-risk populations. Ultimately, we present evidence for the effectiveness of fruit and vegetable intake on subclinical and clinical risk factors for cardiorenal diseases, contributing to the growing field of precision nutrition.

## Figures and Tables

**Table 1:** Baseline Demographic Data and Biometric Characteristics. Baseline demographic, socio-economic, education, and biometric characteristics presented by group (FV intervention and WL control). Missing values range from 0% to 12%. SBP = systolic blood pressure, DBP = diastolic blood pressure, HDL = high-density lipoprotein, LDL = low-density lipoprotein, TC = total cholesterol, TG = triglycerides, BMI = body mass index, HbA1C = hemoglobin A1C, ACR = albumin-to-creatinine ratio.

Variable	All	Intervention (FV)	Control (WL)
N	91	46	45
Age, mean (SD)	58.3 (10.1)	58.5 (8.5)	58.2 (11.6)
Sex, n (%)			
Female	60 (66)	26 (57)	34 (46)
Male	31 (34)	20 (43)	11 (24)
Income, n (%)			
USD0 to less than USD15,000	30 (33.0)	17 (37.0)	13 (28.8)
USD15,000 to less than USD25,000	14 (15.4)	7 (15.2)	7 (15.6)
USD25,000 to less than USD50,000	15 (16.5)	6 (13.0)	9 (20.0)
USD50,000 to 75,000	15 (16.5)	8 (17.4)	7 (15.6)
More than USD75,000	7 (7.6)	3 (6.5)	4 (8.9)
Missing	10 (11.0)	5 (10.9)	5 (11.1)
Education, n (%)			
Some high school	16 (17.6)	8 (17.4)	8 (17.8)
High school diploma	13 (14.3)	7 (15.2)	6 (13.3)
Technical degree	3 (3.3)	1 (2.2)	2 (4.4)
Some college	20 (22.0)	9 (19.6)	11 (24.4)
College degree	28 (30.8)	15 (32.6)	13 (28.9)
Missing	11 (12.0)	6 (13.0)	5 (11.1)
Marital status, n (%)			
Married	27 (29.7)	14 (30.4)	13 (28.9)
Divorced	11 (12.1)	6 (13.0)	5 (11.1)
Separated	4 (4.4)	3 (6.5)	1 (2.2)
Single	32 (35.2)	15 (32.6)	17 (37.8)
Widowed	4 (4.4)	2 (4.3)	2 (4.4)
Living with partner/Unmarried	6 (6.6)	3 (6.5)	3 (6.6)
Missing	7 (7.6)	3 (6.5)	4 (8.9)
Health Outcomes, Mean (SD)			
SBP (mmHg)		136.0 (18.1)	139.4 (19.5)
DBP (mmHg)		82.5 (13.1)	81.8 (12.3)
HDL (mg/dL)		54.9 (18.3)	52.0 (19.1)
LDL (mg/dL)		100.2 (41.4)	92.9 (34.5)
TC (mg/dL)		175.0 (41.3)	168.2 (43.9)
TG (mg/dL)		115.5 (47.5)	118.6 (62.8)
BMI (kg/m^2^)		35.8 (7.3)	34.3 (4.3)
HbA1C (%)		6.7 (1.6)	6.4 (1.2)
ACR (mg/g)		20.8 (22.1)	28.2 (34.0)

**Table 2:** Health Outcomes. Change scores are presented between baseline and 6 weeks post-intervention biometric characteristics by group (FV intervention and WL control). The *p* values indicate the significance in change scores.

Variables (Mean (SD))	Intervention (FV) (6 Weeks—Baseline), n = 38	Control (Waitlist) (6 Weeks—Baseline), n = 30	*p* Value
SBP (mmHg)	−2.9 (16.4)	−2.4 (16.0)	0.89
DBP (mmHg)	0.88 (13.1)	−0.09 (13.3)	0.76
HDL (mg/dL)	1.5 (10.6)	1.7 (7.4)	0.94
LDL (mg/dL)	3.0 (24.0)	2.2 (22.2)	0.91
TC (mg/dL)	−1.3 (23.8)	0.30 (22.4)	0.78
TG (mg/dL)	3.6 (49.8)	−3.0 (51.6)	0.63
BMI (kg/m^2^)	−0.17 (0.96)	−0.25 (0.82)	0.76
HbA1C (%)	0.02 (0.38)	−0.06 (0.42)	0.45
ACR (mg/g)	−5.5 (19.9)	1.8 (24.8)	0.21

## Data Availability

The original contributions presented in the study are included in the article, further inquiries can be directed to the corresponding author. Data will be made available by the corresponding author if requested.
